# 5-Methoxyl Aesculetin Abrogates Lipopolysaccharide-Induced Inflammation by Suppressing MAPK and AP-1 Pathways in RAW 264.7 Cells

**DOI:** 10.3390/ijms17030315

**Published:** 2016-03-01

**Authors:** Lei Wu, Xueqin Li, Haifeng Wu, Wei Long, Xiaojian Jiang, Ting Shen, Qian Qiang, Chuanling Si, Xinfeng Wang, Yunyao Jiang, Weicheng Hu

**Affiliations:** 1 Tianjin Key Laboratory of Pulp & Paper, Tianjin University of Science & Technology, Tianjin 300457, China; wulei858196@163.com; 2 Jiangsu Collaborative Innovation Center of Regional Modern Agriculture & Environmental Protection/Jiangsu Key Laboratory for Eco-Agricultural Biotechnology around Hongze Lake, Huaiyin Normal University, Huaian 223300, China; jiangxj@hytc.edu.cn (X.J.); shenting1019@163.com (T.S.); qiangqian9197@126.com (Q.Q.); 3 Department of Gerontology, Huai’an First People’s Hospital, Nanjing Medical University, Huaian 223300, China; wgc1955@sina.com; 4 Key Laboratory of Bioactive Substances and Resources Utilization of Chinese Herbal Medicine, Ministry of Education, Institute of Medicinal Plant Development, Peking Union Medical College and Chinese Academy of Medical Sciences, Beijing 100193, China; hfwu@implad.ac.cn; 5 Institute of Radiation Medicine, Chinese Academy of Medical Sciences and Peking Union Medical College, Tianjin 300192, China; longway@irm-cams.ac.cn; 6 State Key Laboratory of Tree Genetics and Breeding, Northeast Forestry University, Harbin 150040, China; 7 Department of Medical Biotechnology, College of Biomedical Science, Kangwon National University, Chuncheon 200-701, Korea; yunyao@kangwon.ac.kr

**Keywords:** 5-methoxyl aesculetin, activator protein-1, mitogen-activated protein kinases, inflammation

## Abstract

For the first time, a pale amorphous coumarin derivative, 5-methoxyl aesculetin (MOA), was isolated from the dried bark of *Fraxinus rhynchophylla* Hance (Oleaceae). MOA modulates cytokine expression in lipopolysaccharide (LPS)-treated RAW 264.7 macrophages, but the precise mechanisms are still not fully understood. We determined the effects of MOA on the production of inflammatory mediators and pro-inflammatory cytokines in the LPS-induced inflammatory responses of RAW 264.7 macrophages. MOA significantly inhibited the LPS-induced production of nitric oxide (NO), prostaglandin E_2_ (PGE_2_), tumor necrosis factor-α (TNF-α), interleukin-6, and interleukin-1β. It also effectively attenuated inducible nitric oxide (NO) synthase, cyclooxygenase-2, and TNF-α mRNA expression and significantly decreased the levels of intracellular reactive oxygen species. It inhibited phosphorylation of the extracellular signal-regulated kinase (ERK1/2), thus blocking nuclear translocation of activation protein (AP)-1. In a molecular docking study, MOA was shown to target the binding site of ERK via the formation of three hydrogen bonds with two residues of the kinase, which is sufficient for the inhibition of ERK. These results suggest that the anti-inflammatory effects of MOA in RAW 264.7 macrophages derive from its ability to block both the activation of mitogen-activated protein kinases (MAPKs) and one of their downstream transcription factors, activator protein-1 (AP-1). Our observations support the need for further research into MOA as a promising therapeutic agent in inflammatory diseases.

## 1. Introduction

The Lipopolysaccharide (LPS), an endotoxin from Gram-negative bacteria, is a potent inducer of inflammatory cytokines [1]. LPS-activated macrophages secrete a considerable number of inflammatory mediators, such as nitric oxide (NO), interleukin-6 (IL-6), interleukin-1β (IL-1β), tumor necrosis factor (TNF)-α, and prostaglandin E_2_ (PGE_2_), all of which contribute to host survival following infection and are required for the innate immune response of many mammals [2,3]. In various models of inflammation, inducible NO synthase (*iNOS*) can cause NO production during inflammation, and cyclooxygenase-2 (*COX-2*) is believed to be responsible for the synthesis of PGE_2_ in various models of inflammation [4]. Aberrant resolution and prolonged duration of inflammation have been implicated in the pathophysiology of diseases such as atherosclerosis, rheumatoid arthritis, cerebral malaria, Alzheimer’s disease, Parkinson’s disease, and diabetes [5,6,7]. Accordingly, therapeutic approaches to these inflammatory diseases will necessarily include the modulation of macrophage-mediated inflammatory responses.

LPS binds to toll-like receptor 4, leading to the activation of multiple signaling pathways such as mitogen-activated protein kinases (MAPKs), phosphoinositide 3-kinase (PI3K)/Akt, nuclear factor-kappa B (NF-κB), and Janus kinase-signal transducer and activator of transcription (JAK-STAT) [8,9,10]. MAPKs consist of three subfamilies, whose members include extracellular signal-regulated kinase 1/2 (ERK1/2), p38, and c-Jun N-terminal kinase (JNK). All three proteins play significant roles in regulating macrophage synthesis of inflammatory mediators such as NF-κB and activator protein-1 (AP-1) [11,12]. Conversely, the production of inflammatory mediators is strongly blocked by the suppression of multiple MAPK family members [13,14].

**Figure 1:**
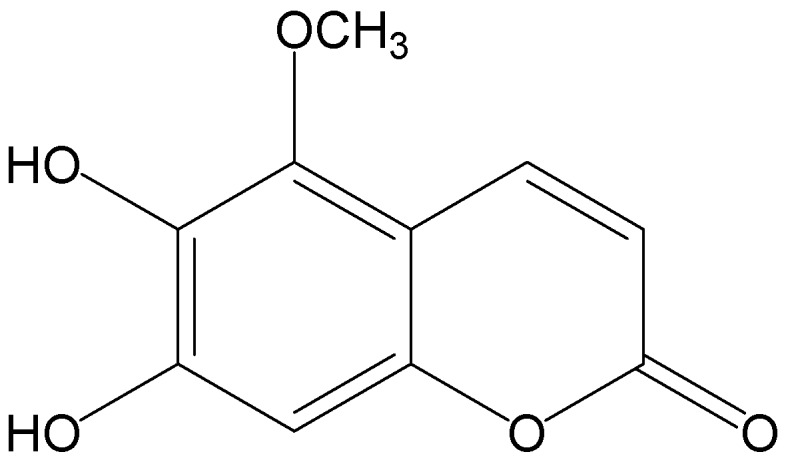
Chemical structure of 5-methoxyl aesculetin (MOA).

*Fraxinus rhynchophylla* (Oleaceae), a deciduous tree widely distributed in China. The bark of *F. rhynchophylla*, well-known as the traditional Chinese herbal drug “Qinpi” listed in Chinese pharmacopoeia, have been used as anti-inflammatory, convergence, febricide, anti-blenophthalmia, anti-diarrhea, and anti-leukorrhea agents for many years [15,16,17]. 5-Methoxyl aesculetin (5-methoxyl aesculetin, MOA) is a pale amorphous coumarin derivative with a molecular weight of 208, the formula C_10_H_8_O_5_, and the chemical structure shown in Figure 1. It was first isolated from the dried bark of *Fraxinus rhynchophylla* Hance (Oleaceae) [17]. Additionally, our HPLC test results revealed that 1 kg of dried bark of *F. rhynchophylla* contained 1202.6 mg MOA. However, the biological activities of MOA are unknown. Therefore, we investigated the anti-inflammatory properties and underlying mechanisms of action of MOA in RAW 264.7 macrophages.

## 2. Results and Discussion

### 2.1. Effect Of 5-Methoxyl Aesculetin (MOA) on the Viability of RAW 264.7 Cells

Cortex Fraxini, the dried bark of *F. rhynchophylla* Hance, has a variety of biological activities, including antioxidant, anticancer, anti-inflammatory, and photo-protective effects [18,19,20,21]. A large number of coumarins exhibit anti-inflammatory properties, including isofraxidin, which inhibits TNF-α production by LPS-induced mouse peritoneal macrophages via the MAPK pathway [22]; IMM-H004, which attenuates the production of inflammatory mediators in LPS-stimulated BV2 microglia [23]; and psoralidin, which inhibits LPS-induced *iNOS* expression by repressing Syk-mediated activation of PI3K-IKK-IκB signaling pathways [24]. Previous phytochemical studies isolated the coumarins ferulaldehyde, scopoletin, fraxidin, fraxetin, aesculetin, aesculin, fraxin, 6,7-dimethoxy-8-hydroxycoumarin, and fraxisecoside [25,26,27]. In our previous investigation, four coumarins, including umbelliferone, aesculetin, MOA, and aesculin, were isolated from the bark of *F. rhynchophylla*. Earlier studies suggested that umbelliferone possessed strong anti-inflammatory activities [17,28], and it was reported that aesculetin and 4-methylesculetin might be effective for the treatment of intestinal inflammatory bowel disease [29]. Meanwhile, aesculin was concluded to be a potent drug candidate that protected against the inflammatory destruction [30]. Our group was the first to isolate MOA from the dried bark of *F. rhynchophylla* Hance, but the biological activities of this coumarin have yet to be explored.

**Figure 2:**
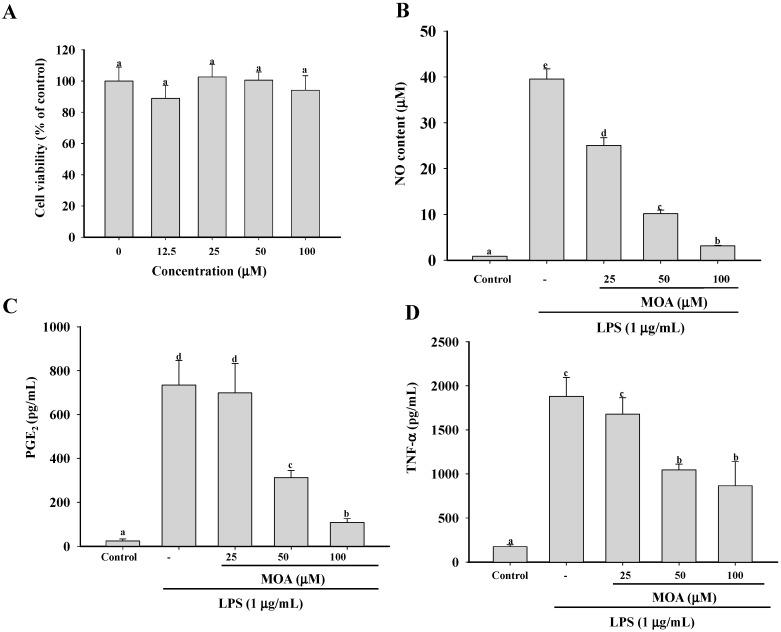

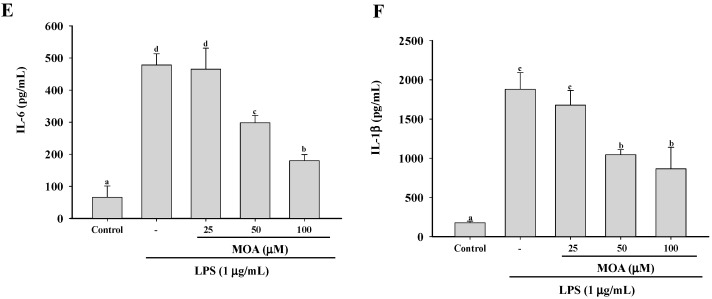
Effects of MOA on cell viability and pro-inflammatory cytokine production in lipopolysaccharide (LPS)-induced RAW264.7 cells. The cells were treated with 12.5, 25, 50, or 100 μM MOA for 24 h at 37 °C, after which cell viability was determined by MTT (**A**) assay. The cells were also incubated with 12.5, 25, 50, or 100 μM MOA for 30 min and then with or without LPS (1 μg/mL) for 24 h; the supernatants were collected for measurements of (**B**) nitric oxide (NO); (**C**) prostaglandin E_2_ (PGE_2_); (**D**) tumor necrosis factor-α (TNF-α); (**E**) interleukin-6 (IL-6); and (**F**) interleukin-1β (IL-1β) production using Griess reagent (NO) and enzyme immunoassay kits (all other cytokines). The experiments were performed in triplicate. The data are expressed as means ± standard deviations (SDs) (*n* = 3). Values with the same lower case superscript letters are not significantly different from each other at *p* < 0.05.

Because the anti-inflammatory effect of MOA may be related to its cytotoxicity, it was important in this study to first determine the subcytotoxic concentrations. To do so, we incubated RAW 264.7 cells with various concentrations of MOA for 24 h and then determined cell viability using 1-(4,5-dimethylthiazol-2-yl)-3,5-diphenylformazan (MTT) assay. As shown in Figure 2A, the viability of RAW 264.7 cells was not significantly affected by a 24-h incubation with 12.5, 25, 50, and 100 µM MOA, whereas higher concentrations caused significant cytotoxicity (data not shown). Therefore, in the next experiments, nontoxic concentrations of MOA were used, which ruled out the possibility that the effect of MOA was due to a reduction in cell viability.

### 2.2. Effect of MOA on Nitric Oxide (NO) Production and Pro-Inflammatory Cytokine Production

Macrophages secrete NO and pro-inflammatory cytokines in response to bacterial LPS [31]. NO is a gaseous cellular signaling molecule involved in many inflammatory processes. Its production is regulated by three isoforms of NOS: neuronal NOS (*nNOS*), endothelial NOS (*eNOS*), and *iNOS* [32]. Both nNOS and eNOS are activated by Ca^2+^/calmodulin, but *iNOS* is expressed by macrophages in response to inflammatory stimuli such as cytokines and some pathogens; it does not depend on Ca^2+^ [33]. *COX* is an important mediator in inflammation because of its role in prostaglandin biosynthesis [34]. Its two isoforms, *COX-1* and *COX-2*, produce the same products and catalyze the same reaction using identical catalytic mechanisms, but the two enzymes differ in their inhibitor selectivity. *COX-1* appears to be responsible for maintaining homeostasis, whereas *COX-2* is transiently induced by growth factors, cytokines, chemokines, and bacterial toxins [35]. TNF-α is perhaps the most well studied of the first-line cytokines, which aggravate and prolong inflammatory injury by inducing autoimmune reactions, specifically, the activation of T cells and macrophages. The mechanism underlying the activity of TNF-α is the upregulation of other pro-inflammatory cytokines, which in turn enhance the recruitment of leukocytes to the site of inflammation. In addition, TNF-α induces the release of IL-1β and IL-6, thereby enhancing the sensitivity of tissue macrophages to this cytokine [36]. Thus, the inhibition of NO, PGE_2_, and TNF-α production, by blocking their mRNA expression, may be a useful strategy for the treatment of various inflammatory disorders. In the present study, to investigate the pharmacological effects of MOA on the production of pro-inflammatory cytokines in RAW 264.7 cells and the underlying mechanisms, non-cytotoxic concentrations of MOA were added to the cells in the absence and presence of LPS. After 24 h, NO production was determined using the Griess reagent to measure the amount of nitrite (a stable oxidized product of NO) released into the medium upon LPS stimulation. The concentration of NO in the medium of the LPS-stimulated cells after 24 h increased significantly, by approximately 45-fold (39.53 μM), compared to the untreated cells (0.87 μM) (Figure 2B). However, in the presence of 25, 50, and 100 μM MOA, NO production by LPS-stimulated cells was significantly reduced, to 25.08, 10.19, and 3.17 μM, respectively. This result is similar to the effect observed with NG-nitro-l-arginine methyl ester (l-NMA), an inhibitor of NO production (data not shown). Jonville *et al.* (2011) [37] also found that the ethyl acetate-extracted fraction of the edible brown alga *Saccharina japonica* suppressed NO production (IC_50_ = 25.32 μg/mL) in LPS-induced RAW264.7 macrophages. In Reyes *et al.* (2006) [38], acacetin, isolated from *Cirisium rhinoceros* Nakai, inhibited NO in a dose-dependent manner, with an IC_50_ of 13.78 μM.

The stimulation of RAW 264.7 with LPS for 24 h resulted in a significant increase in PGE_2_, TNF-α, IL-1β, and IL-6 production (Figure 2C–F). Moreover, pretreatment with MOA markedly inhibited pro-inflammatory cytokine release in a concentration-dependent manner. Taken together, these results indicate that MOA inhibits the initial phase of the inflammatory cascade by suppressing LPS-mediated PGE_2_, TNF-α, IL-1β, and IL-6 secretion.

### 2.3. Effects of MOA on the Expression Levels of Inducible NO Synthase (iNOS), Cyclooxygenase-2 (COX-2), and TNF-α mRNA in RAW 264.7 Cells

**Figure 3:**
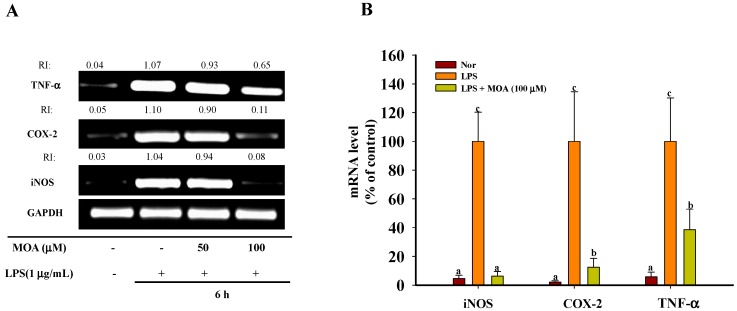
Effects of MOA on LPS-induced inducible NO synthase (*iNOS*), cyclooxygenase-2 (*COX-2*), and *TNF-α* expression by RAW 264.7 cells. After pretreatment with the indicated concentrations of MOA for 30 min, LPS (1 μg/mL) was added, and the cells were incubated for a further 6 h. The levels of *TNF-α*, *COX-2*, *iNOS*, and glyceraldehyde 3-phosphate dehydrogenase (GAPDH) mRNA were determined by semi-quantitative polymerase chain reaction (PCR) (**A**) and real-time PCR (**B**). The experiment was repeated three times, and similar results were obtained. The data are expressed as the means ± SDs (*n* = 3). Values with the same lower case superscript letters are not significantly different from each other at *p* < 0.05.

To determine whether the MOA-mediated inhibition of NO formation involved modulations of *iNOS, COX-2*, and *TNF*-α gene expression, RAW 264.7 cells that were pretreated with the indicated concentrations of MOA for 30 min and then stimulated with LPS (1 μg/mL) for 6 h were subjected to RT-PCR analysis for *iNOS* and *COX-2* mRNA. As shown in Figure 3A,B, both *iNOS* and *COX-2* mRNAs were undetectable in RAW 264.7 cells that were incubated in the medium alone, whereas their expression was markedly upregulated in response to LPS; however, mRNA levels were strongly inhibited by MOA in a concentration-dependent manner. There were no effects on the expression of the housekeeping gene *GAPDH*. These results suggest that, in LPS-stimulated macrophages, MOA directly suppresses NO and PGE_2_ by inhibiting the expression of *iNOS* and *COX-2*.

### 2.4. Effects of MOA on Free Radicals and Intracellular Reactive Oxygen Species (ROS) Levels

**Figure 4:**
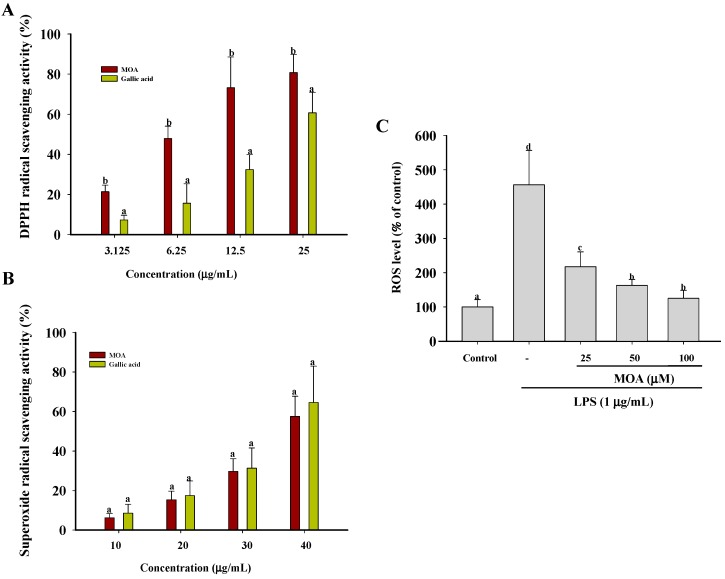
Effects of MOA on free radical production and LPS-induced intracellular reactive oxygen species (ROS) production. (**A**) Free radical-scavenging activity of MOA as determined in a 1,1-diphenyl-2-picrylhydrazyl (DPPH) assay. Gallic acid served as the positive control; (**B**) Superoxide radical-scavenging activity of MOA. Gallic acid served as the positive control; (**C**) The cells were pretreated with different concentrations of MOA for 30 min and then with LPS (1 μg/mL) for 6 h. Intracellular ROS levels were measured based on 2,7-dichlorofluorescin diacetate fluorescence. The experiments were performed in triplicate. The data are expressed as means ± SDs (*n* = 3). Values within a column that have the same lower case superscript letters are not significantly different from each other at *p* < 0.05.

The imbalance between ROS generation and defense mechanisms leads to the oxidative damage of biological molecules and therefore to free radical-mediated pathologies [39]. The antioxidant activities of natural products must be determined by several different methods, as a single assay able to test all of these compounds is currently not available [40]. MOA revealed better 1,1-diphenyl-2-picrylhydrazyl (DPPH) free radical scavenging activity and superoxide radical scavenging activity (Figure 4A,B). This activity can be attributed to the 6,7-dihydroxyl of coumarin, given that structures with free hydroxyl groups act as efficient scavengers of free radicals.

Inflammation is in part mediated by oxidative stress. LPS, a major component of the outer membrane of Gram-negative bacteria, causes severe sepsis and syndromes related to the resulting multiple organ dysfunction. The body’s response to LPS includes the generation of ROS and the secretion of pro-inflammatory cytokines and other mediators, such as NO, TNF-α, IL-1β, IL-6, and PGE_2_, by macrophages [41]. Previous literature reveals that inhibitory effects on MAPK, NF-κB, and AP-1 signaling are related to a decrease in ROS [42,43]. Therefore, we examined the ability of MOA to attenuate the LPS-induced intracellular accumulation of ROS. As shown in Figure 4C, at concentrations of 25, 50, and 100 μM, MOA significantly decreased LPS-stimulated ROS production to 217.43%, 163.23%, and 125.21%, respectively. These results demonstrate that the antioxidative properties of MOA inhibit the intracellular production of ROS induced by LPS.

### 2.5. Effects of MOA on Lipopolysaccharide (LPS)-Induced Mitogen-Activated Protein Kinases (MAPK)/Activator Protein-1 (AP-1) Activation in RAW 264.7 Cells

**Figure 5:**
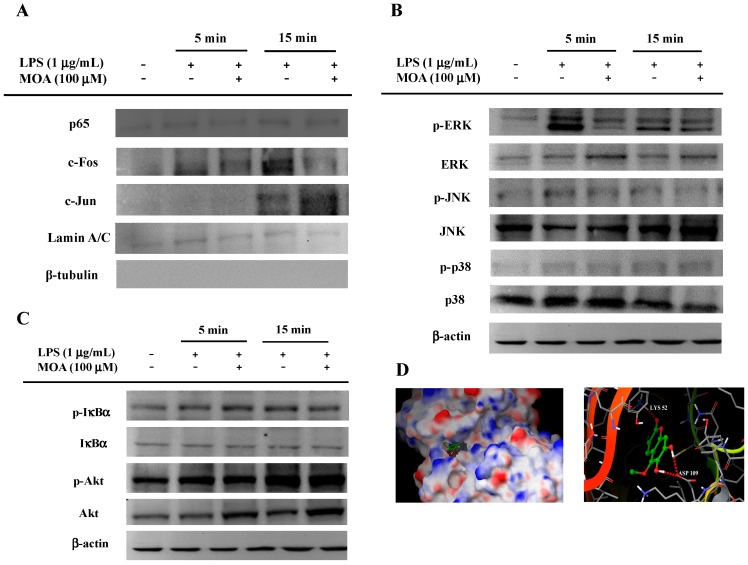
Effects of MOA on the activation of the upstream signaling pathways for activator protein-1 (AP-1) translocation. RAW264.7 cells (5 × 10^6^ cells/mL) pretreated with MOA for 30 min were stimulated in the absence or presence of LPS (1 μg/mL) for an additional 5 or 15 min. (**A**) The translocated levels of c-Jun, c-Fos, p65, lamin A/C, and β-tubulin were determined by immunoblotting of the nuclear fraction of the cells; whole-cell lysates were extracted for immunoblotting to measure the levels of (**B**) phospho- or total MAPKs (extracellular signal-regulated kinase (ERK), p38, and c-Jun N-terminal kinase (JNK)) or (**C**) IκBα and Akt; (**D**) superposition of the crystal structure of ERK with the docking structure of MOA. Hydrogen bonds between compound and amino acids are shown by dotted lines. All experiments were repeated three times. Representative results are shown.

The activation of transcriptional factors such as NF-κB and AP-1 is involved in inflammation [44]. Upon stimulation, cytoplasmic NF-κB and AP-1 translocate to the nucleus, where they mediate the expression of many pro-inflammatory genes [45]. The transcriptional control of pro-inflammatory mediators requires the activation of redox-sensitive transcription factors in LPS-stimulated macrophages [46]. The promoters of the *iNOS* and *COX-2* genes contain several homologous consensus sequences for the binding of NF-κB- and AP-1 [47]. To examine the nuclear translocation of AP-1 and NF-κB in LPS-treated RAW264.7 cells, nuclear fractions were prepared and analyzed by immunoblotting. As shown in Figure 5A, MOA did not affect the nuclear level of p65, a major NF-κB subunit, in LPS-treated *vs.* untreated conditions. However, MOA did inhibit the LPS-induced phosphorylation of c-Fos, required for nuclear translocation, but had no effect on the nuclear translocation or phosphorylation of c-Jun. The nuclear fractions were also determined to be free from cytoplasmic contamination. Previous literature reveals that numerous compounds such as α-chaconine, quercetin, costunolide, and 2’-benzoyloxycinnamaldehyde inhibit NO production through AP-1 signaling pathway, indicating that the inhibition of AP-1 could be a general feature of anti-inflammatory remedies [48,49,50,51]. IκBα phosphorylation, a critical event for NF-κB translocation, was not inhibited by MOA, indicating that the NF-κB pathway is not responsive to MOA exposure (Figure 5C). Because AP-1 translocation is mainly regulated by MAPKs such as p38, JNK, and ERK, we investigated the effects of MOA on these three kinases. MOA inhibited the LPS-induced phosphorylation of ERK, but not of p38 or JNK (Figure 5B). To assess the interaction between MOA and ERK in detail, we examined the 3D structure of the protein using information obtained from the Protein Data Bank (2OJG) and identified the potential MOA-binding sites through a molecular docking study. As depicted in Figure 5D, MOA inhibits ERK by forming three hydrogen bonds with two residues, Lys-52 and Asp-109, in the active site (G-score of −6.71). Taken together, these results suggest that MOA suppresses the LPS-induced expression of *iNOS*, *COX-2*, and *TNF-α* by inhibiting the phosphorylation of ERK in RAW 264.7 cells.

## 3. Materials and Methods

### 3.1. Chemicals and Reagents

MOA (Figure 1, molecular weight: 208 for C_10_H_8_O_5_, purity: 99.6%) was isolated and purified from the dried bark of *F. rhynchophylla* Hance as described previously [17]. 1,1-Diphenyl-2-picrylhydrazyl (DPPH), gallic acid, nitro blue tetrazolium (NBT), 2,7-dichlorofluorescin diacetate (DCFH-DA), phenazine methosulphate (PMS), 1-(4,5-dimethylthiazol-2-yl)-3,5-diphenylformazan (MTT), sulfanilamide, naphthylethylenediamine dihydrochloride, LPS (*Escherichia coli* 0111:B4), NG-monomethyl-l-arginine (l-NMA), and β-nicotinamide adenine dinucleotide (NADH) were from Sigma (St. Louis, MO, USA). The kits for RNA isolation and first-strand cDNA synthesis were obtained from Invitrogen (Carlsbad, CA, USA) and SYBR Premix Ex Taq was from Takara Bio (Shiga, Japan). The Roswell Park Memorial Institute (RPMI) medium 1640, trypsin-EDTA, and antibiotics were from Gibco BRL (Life Technologies, Shanghai, China). Fetal bovine serum (FBS) was from Gibco BRL (Grand Island, NY, USA). Primary antibodies against phospho-specific ERK, c-Jun N-terminal kinase (JNK), IκBα, Akt, p38, and all antibodies to ERK, JNK, IκBα, Akt, p38, and β-actin were from Cell Signaling Technology (Beverly, MA, USA). Antibodies against LaminA/C and β-tubulin were purchased from Santa Cruz Biotechnology (Santa Cruz, CA, USA). Horseradish peroxidase-conjugated goat anti-rabbit and goat anti-mouse antibodies were provided by Abcam (Cambridge, MA, USA). Antibody binding was measured using the enhanced chemiluminescence (ECL) substrate (ComWin Biotech, Beijing, China). ELISA kits for PGE_2_, TNF-α, IL-6, and IL-1β were from R & D Systems (Minneapolis, MN, USA). All other chemicals were of analytical grade.

### 3.2. Cell Line and Cell Culture

RAW 264.7 murine macrophages were obtained from the American Type Culture Collection (Manassas, VA, USA) and cultured in the RPMI 1640 medium supplemented with l-glutamine (2 mM), 10% inactivated FBS, 100 U penicillin/mL, and 100 µg streptomycin/mL (Biological Industries, Bet Haemek, Israel). The cells were incubated at 37 °C in a humidified 5% CO_2_ incubator (MCO-15AC CO_2_ incubator, SANYO, Osaka, Japan). The medium was changed routinely every 2 days. Confluent RAW 264.7 cells were passaged by scraping them with a sterile cell scraper.

### 3.3. Cell Viability Assay

The cytotoxicity of MOA for RAW 264.7 cells was investigated using the MTT assay [52]. The cells were seeded in 96-well flat-bottom culture plates at a density of 1 × 10^5^ cells/well, incubated overnight at 37 °C, and then treated with the indicated concentrations of MOA for 24 h. After the supernatants had been carefully aspirated from each well, a 10-µL MTT stock solution and a 90-µL FBS-free medium were added to each well to achieve a total volume of 100 µL. The plates were incubated for 4 h at 37 °C, after which the formazan crystals that had formed were solubilized with the 100 µL-MTT stop solution. The amount of purple formazan was measured at 550 nm using an Infinite M200 Pro spectrophotometer (Tecan, Männedorf, Switzerland). The data are expressed as the percentage of the control optical density (OD) values for each experiment.

### 3.4. Determination of NO, Prostaglandin E_2_ (PGE_2_), Tumor Necrosis Factor-α (TNF-α), Interleukin-1β (IL-1β), and IL-6 Production

NO was measured based on the detection of its stable oxidative metabolite via the Griess reaction, as previously described [53]. Briefly, RAW 264.7 cells (1 × 10^5^ cells/well) were plated in 96-well flat-bottom culture plates and incubated overnight at 37 °C. The medium was removed and the cells were further incubated for 30 min in the absence or presence of various concentrations of MOA or with l-NMA (positive control). Then, they were stimulated with LPS (1 µg/mL) for an additional 24 h. Next, a 100-µL culture medium was mixed with an equal volume of Griess reagent and transferred to a 96-well plate. After a 5-min incubation at room temperature, the absorbance of the samples at 550 nm was measured using an Infinite M200 Pro spectrophotometer. A fresh culture medium was used as a blank in every experiment. The nitrite concentration was determined using a calibration curve based on a sodium nitrite standard curve. The concentrations of PGE_2_, TNF-α, l-6, and IL-1β released in the supernatant were measured using enzyme immunoassay kits according to the manufacturer’s instructions.

### 3.5. RNA Extraction and Reverse Transcription-Polymerase Chain Reaction

RAW 264.7 cells were seeded at 5 × 10^6^ cells in 60-mm cell culture plates with a 4-mL culture medium and incubated for 16 h at 37 °C. Then, the cells were treated first with different concentrations of MOA for 30 min and then with LPS for an additional 6 h. Total RNA was isolated using the Trizol reagent (Invitrogen). The concentration and purity of the RNA were measured by agarose gel electrophoresis and a UV spectrophotometer (Nanodrop 2000c, Thermo Scientific, Wilmington, DE, USA). To obtain cDNA by reverse transcription, 2 μg of total RNA was incubated with an oligo (dT) 15-mer primer and dNTPs for 5 min at 65 °C. Then, the reaction tubes were placed on ice, and a 5× first-strand buffer, an RNase inhibitor, and 0.1 M dl-dithiothreitol (DTT) were added. The reaction mixture was further incubated for 2 min at 37 °C and then again for 1 h after adding moloney murine leukemia virus (M-MLV) reverse transcriptase. The reaction was stopped by heating at 70 °C. The primer sequences and conditions used in the PCRs are listed in Table 1. The PCR products were electrophoresed on 1% agarose gels and visualized using ethidium bromide. The intensities of the bands in the digitally imaged gels were determined using a Gel Doc XR system (Bio-Rad, Hercules, CA, USA). mRNA was quantified in real-time RT-PCR using SYBR Premix Ex Taq (TaKaRa, Dalian, China) and a real-time thermal cycler (Bio-Rad), according to the manufacturer’s instructions.

A dissociation curve analysis of *iNOS*, *COX-2*, *TNF-α*, and the housekeeping gene glyceraldehyde 3-phosphate dehydrogenase (*GAPDH*) showed a single peak for each compound. The real-time PCR conditions were as follows: 94 °C for 10 min followed by 30 cycles at 94 °C for 15 s, 55 °C for 15 s, and 72 °C for 30 s, with a final extension at 72 °C for 1 min. The relative expression levels of iNOS, *COX-2*, *TNF-α*, and *GAPDH* were calculated from triplicate measurements and normalized to the mean Ct of *GAPDH*.

### 3.6. 1,1-Diphenyl-2-picrylhydrazyl (DPPH) Radical-Scavenging Activity

The free-radical scavenging activity of MOA was analyzed *in vitro* using the DPPH radical as previously described [54]. Briefly, 0.1 mL 0.2 mM DPPH (in methanol) was placed in each well of a 96-well plate to which serial dilutions of MOA were added. Then, the plate was shaken vigorously and left to stand for 30 min in the dark. Gallic acid was used as a reference antioxidant. Discoloration of the reaction mixture was measured at 517 nm using an Infinite M200 Pro spectrophotometer (Tecan).

### 3.7. Superoxide-Radical Scavenging Assay

Superoxide-radical scavenging activity was measured using the NBT reduction method [55]. The superoxide radicals were generated in phosphate buffer (0.1 M, pH 7.4) containing 100 μL of various concentrations of MOA, 150 μL NADH (166 μM), 450 μL NBT (86 μM), and 150 μL PMS (16.2 μM). After a 5-min incubation at room temperature, the absorbance of the treated samples at 560 nm was measured against that of the blank samples. A decrease in the absorbance of the reaction mixture indicated increased superoxide-anion scavenging activity.

### 3.8. Intracellular ROS Inhibition Activity

RAW 264.7 cells (1 × 10^5^ cells/well) were plated in a 96-well black plate and incubated overnight at 37 °C. Then, the medium was removed and the cells were treated (or not) with various concentrations of MOA for 30 min and then with LPS (1 µg/mL). After 24 h, the supernatant of each well was removed, and the cells were washed twice with PBS. DCFH-DA (10 μM) was added to each well and the cells were incubated for 20 min at 37 °C. Then, the DCFH-DA was replaced with 100 μL cold PBS. ROS concentrations were measured using an Infinite M200 Pro spectrophotometer (Tecan). The excitation wavelength was 488 nm, and the emission wavelength was 535 nm.

### 3.9. SDS-PAGE and Western Blot Analysis

RAW 264.7 cells were seeded at 5 × 10^6^ cells in 60 mm cell culture plates containing 4 mL culture medium and incubated for 16 h at 37 °C. Then, they were treated first with MOA for 30 min and afterwards with LPS for the indicated time. The cells were washed twice with PBS and collected by centrifugation. The washed cell pellets were resuspended in a 200-μL lysis buffer (ComWin Biotech, Beijing, China) and a Roche Complete protease inhibitor cocktail (Roche Diagnostics Ltd., Mannheim, Germany). The cell lysates were centrifuged at 12,000× *g* for 5 min at 4 °C. The supernatant was collected and the protein concentration was measured using the bicinchoninic acid (BCA) protein assay kit (ComWin Biotech). Then, nuclear protein was extracted from the cells using a nuclear protein isolation kit (ComWin Biotech). To quantify the expression of the proteins of interest, equal amounts of protein were boiled at 95 °C for 5 min, subjected to 10% sodium dodecyl sulfate (SDS)-polyacrylamide gel electrophoresis (PAGE), and transferred to polyvinylidene fluoride (PVDF) membranes. The membranes were blocked in a blocking buffer for 60 min at room temperature and then incubated with the indicated primary antibody for 1 h at room temperature. After three washes with a TBST buffer, they were incubated for 60 min with secondary antibodies conjugated to horseradish peroxidase (HRP)-conjugated goat immunoglobulin G and washed three times with TBST buffer. Bound antibodies were detected using the enhanced chemiluminescence (ECL) system, and the reaction products were detected with the Tanon-5200 chemiluminescence detection system (Tanon Science, Shanghai, China).

### 3.10. Molecular Docking Study of MOA

The molecular structure of MOA was drawn using Maestro 8.5 (Schrodinger Suite 2008, Schrödinger, Limited Liability Company (LLC), New York, NY, USA), which was also used to energy-minimize the structures in the final models. The 3D structures of ERK were downloaded from Protein Data Bank (PDB, ID: 2OJG) (Avaliable online: http://www.rcsb.org/pdb/home/home.do). Protein structures were fixed and optimized using Protein Preparation Wizard (Schrodinger Suite 2008) and then used for molecular docking using Glide 5.0 (Schrodinger Suite 2008) in standard precision (SP) mode with default parameter settings. All of the calculations were performed on a Dell workstation with 8 Xeon^®^ CPUs (Intel, Santa Clara, CA, USA).

### 3.11. Data Analysis

One-way analysis of variance (ANOVA) was used to determine significant differences between groups, followed by a Duncan’s multiple range test or Student’s t-test. All experiments were carried out in triplicate (*n* = 3). The values are expressed as the means of three replicate determinations ± standard derivation (SD). Differences between groups were considered significant at *p* < 0.05. All analyses were performed using SPSS for Windows 7, version 20 (SPSS Inc., Chicago, IL, USA).

## 4. Conclusions

This study clearly demonstrated the potent anti-inflammatory activities of MOA from Cortex Fraxini and the ability of this coumarin to inhibit the secretion of inflammatory cytokines, including NO, PGE_2_, TNF α, IL-1β, and IL-6, following LPS stimulation. MOA also inhibited the transcription of *iNOS*, *COX-2*, and *TNF-α*. Our results suggest that the mechanism underlying the effects of MOA on inflammatory mediators involves the prevention of AP-1 nuclear translocation through the downregulation of ERK1/2 in the LPS-activated MAPK pathway of RAW 264.7 cells. Our findings support the need for further studies examining the therapeutic potential of MOA for the prevention and treatment of inflammatory diseases.

## Figures and Tables

**Table 1:** This Primer sequences and conditions for RT-PCR.

Gene Name	Primer Sequence (5′-3′)	PCR Conditions	PCR Cycles
*GAPDH*	F: CACTCACGGCAAATTCAACGGCA	Denaturation-94 °C, 30 s	30
	R: GACTCCACGACATACTCAGCAC	Annealing-60 °C, 30 s	
		Extension-72 °C, 30 s	
*iNOS*	F: CCCTTCCGAAGTTTCTGGCAGCAG	Denaturation-94 °C, 30 s	27
	R:GGCTGTCAGAGCCTCGTGGCTTTGG	Annealing-60 °C, 30 s	
		Extension-72 °C, 30 s	
*COX-2*	F: CACTACATCCTGACCCACTT	Denaturation-94 °C, 30 s	30
	R: ATGCTCCTGCTTGAGTATGT	Annealing-55 °C, 30 s	
		Extension-72 °C, 30 s	
*TNF-α*	F: TGCCTATGTCTCAGCCTCTTC	Denaturation-94 °C, 30 s	30
	R: GAGGCCATTTGGGAACTTCT	Annealing-55 °C, 30 s	
		Extension-72 °C, 30 s	

## Abbreviations

LPS	Lipopolysaccharide
NO	Nitric oxide
TNF-α	Tumor necrosis factor-α
NF-κB	Nuclear factor-kappa B
AP-1	Activator protein-1
MOA	5-Methoxyl aesculetin
ECL	Enhanced chemiluminescence
PBS	Phosphate-buffered saline
PGE_2_	Prostaglandin E2
ERK1/2	Extracellular signal-regulated kinase
MAPKs	Mitogen-activated protein kinases
PI3K	Phosphoinositide 3-kinase
iNOS	Inducible nitric oxide synthase
DPPH	1,1-Diphenyl-2-picrylhydrazyl
NBT	Nitro blue tetrazolium
DCFH-DA	2,7-Dichlorofluorescin diacetate
PMS	Phenazine methosulphate
MTT	1-(4,5-Dimethylthiazol-2-yl)-3,5-diphenylformazan
l-NMA	NG-monomethyl-l-arginine
GAPDH	Glyceraldehyde 3-phosphate dehydrogenase
